# A Highly Prevalent and Pervasive Densovirus Discovered among Sea Stars from the North American Atlantic Coast

**DOI:** 10.1128/AEM.02723-19

**Published:** 2020-03-02

**Authors:** Elliot W. Jackson, Charles Pepe-Ranney, Mitchell R. Johnson, Daniel L. Distel, Ian Hewson

**Affiliations:** a Department of Microbiology, Cornell University, Ithaca, New York, USA; b AgBiome, Inc., Research Triangle Park, North Carolina, USA; c Ocean Genome Legacy Center of New England BioLabs, Northeastern University Marine Science Center, Nahant, Massachusetts, USA; University of Tennessee at Knoxville

**Keywords:** densovirus, echinoderm, metaviromics, microbiome, parvovirus, sea star, viral discovery, viral metagenomics

## Abstract

The etiology of sea star wasting syndrome is hypothesized to be caused by a densovirus, sea star-associated densovirus (SSaDV), that has previously been reported on the Pacific and Atlantic Coasts of the United States. In this study, we reevaluated the presence of SSaDV among sea stars from the North American Atlantic Coast and in doing so discovered a novel densovirus that we have named *Asterias forbesi*-associated densovirus (AfaDV), which shares 78% nucleotide pairwise identity with SSaDV. In contrast to previous studies, SSaDV was not detected in sea stars from the North American Atlantic Coast. Using a variety of PCR-based techniques, we investigated the tissue tropism, host specificity, and prevalence of AfaDV among populations of sea stars at five locations along the Atlantic Coast. AfaDV was detected in three sea star species (Asterias forbesi, Asterias rubens, and *Henricia* sp.) found in this region and was highly prevalent (>80% of individuals tested; *n* = 134), among sampled populations. AfaDV was detected in the body wall, gonads, and pyloric caeca (digestive gland) of specimens but was not detected in their coelomic fluid. A significant difference in viral load (copies mg^−1^) was found between tissue types, with the pyloric caeca having the highest viral loads. Further investigation of *Asterias forbesi* gonad tissue found germ line cells (oocytes) to be virus positive, suggesting a potential route of vertical transmission. Taken together, these observations show that the presence of AfaDV is not an indicator of sea star wasting syndrome because AfaDV is a common constituent of these animals’ microbiome, regardless of health.

**IMPORTANCE** Sea star wasting syndrome is a disease primarily observed on the Pacific and Atlantic Coasts of North America that has significantly impacted sea star populations. The etiology of this disease is unknown, although it is hypothesized to be caused by a densovirus, SSaDV. However, previous studies have not found a correlation between SSaDV and sea star wasting syndrome on the North American Atlantic Coast. This study suggests that this observation may be explained by the presence of a genetically similar densovirus, AfaDV, that may have confounded previous studies. SSaDV was not present in sea stars screened in this study, and instead, AfaDV was commonly found in sea star populations across the New England region, with no apparent signs of disease. These results suggest that sea star densoviruses may be common constituents of the animals’ microbiome, and the diversity and extent of these viruses among wild populations may be greater than previously recognized.

## Abstract (precis)

Sea star wasting syndrome is a disease primarily observed on the Pacific and Atlantic Coasts of North America that has significantly impacted sea star populations. The etiology of this disease is unknown, although it is hypothesized to be caused by a densovirus, SSaDV. However, previous studies have not found a correlation between SSaDV and sea star wasting syndrome on the North American Atlantic Coast. This study suggests that this observation may be explained by the presence of a genetically similar densovirus, AfaDV, that may have confounded previous studies. SSaDV was not present in sea stars screened in this study, and instead, AfaDV was commonly found in sea star populations across the New England region, with no apparent signs of disease. These results suggest that sea star densoviruses may be common constituents of the animals’ microbiome, and the diversity and extent of these viruses among wild populations may be greater than previously recognized.

## INTRODUCTION

Densoviruses, also known as densonucleosis viruses, are icosahedral, nonenveloped viruses that have monopartite linear single-stranded DNA (ssDNA) genomes that are typically 4 to 6 kb packaged in a 20- to 25-nm-diameter capsid shell (1, 2). Densoviruses belong to the subfamily *Densovirinae*, which is part of the *Parvoviridae* family, and are known to infect arthropods, specifically insects and shrimp (1). Prior to the advent of high-throughput sequencing technology, the discovery of densoviruses was driven by investigations of epizootics occurring in laboratory populations and breeding facilities for economically important invertebrates (e.g., silkworms, crickets, and shrimp) or through infected cell lines (e.g., mosquito C6/36 cell line) (3–7). The majority of densoviruses isolated to date share a pathology, causing hypertrophied nuclei in affected tissues, and are generally more virulent at early life stages of their host (1). Densoviruses have also been shown to be mutualists. For example, sublethal infections in rosy apple aphids are correlated with a winged phenotype that has a lower fecundity than nonwinged aphids (8). The cost of infection lowers the fecundity of the individual to promote the growth of wings that increase mobility and the potential for the host and the virus to disperse (8). Although densoviruses have been primarily studied in insects and crustaceans, analyses of transcriptomic data sets and viral metagenomes prepared from metazoan tissues have found endogenous and exogenous densovirus sequences from a much wider host range (9–13). These findings suggest that densoviruses may be common constituents of many invertebrate viromes.

Densovirus sequences have recently been recovered from echinoderm tissues and have been implicated as potential pathogens, although their relationship to echinoderms is unknown (10, 11, 14). From 2013 to 2015, a mortality event termed sea star wasting disease or syndrome (SSWS) (also referred to as asteroid idiopathic wasting syndrome) affected >20 sea star species along a broad geographic range from California to Alaska. A densovirus (sea star-associated densovirus [SSaDV]) was discovered and hypothesized to cause SSWS (11, 14). Previous work reported the presence of SSaDV, using PCR and quantitative PCR (qPCR) (11, 15, 16), among sea stars on the North American Atlantic Coast, although it was not found to be significantly correlated with SSWS (15, 16). The cause of SSWS among echinoderms on the Atlantic Coast is, however, hypothesized to be viral in nature (16).

We sought to reinvestigate the presence of SSaDV and more generally survey the viral diversity of densoviruses in sea stars inhabiting the North American Atlantic Coast. Viral metagenomes were prepared from Asterias forbesi, a common sea star found in the subtidal environment from the North American Atlantic Coast, which led to the discovery of a novel sea star densovirus referred to here as *Asterias forbesi*-associated densovirus (AfaDV). AfaDV is the second sea star-associated densovirus to be discovered in sea stars thus far. In contrast to previous work, we did not find any evidence of SSaDV among sea stars on the Atlantic Coast through metagenomic analysis and PCR surveys (11, 15, 16). Using a variety of PCR techniques, we investigated the geographic distribution, the host specificity, the tissue tropism, and the potential for vertical transmission of AfaDV. Our results show that AfaDV has a broad geographic range, is not species specific, has a wide tissue tropism, and is potentially vertically transmitted.

## RESULTS

### Genome analysis and phylogeny.

**FIG 1:**
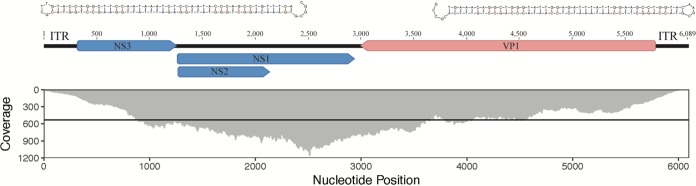
Genome architecture and base coverage of *Asterias forbesi*-associated densovirus (AfaDV). (Top) Structural hairpins 88 nt long located in the inverted terminal repeats at the end of the genome. (Middle) Genome organization with ORFs colored by putative function. Red corresponds to the structural protein (VP), and blue corresponds to nonstructural proteins (NS1, NS2, and NS3). (Bottom) Read coverage distribution across the genome. The black line indicates 532× average base coverage across the genome.

**FIG 2:**
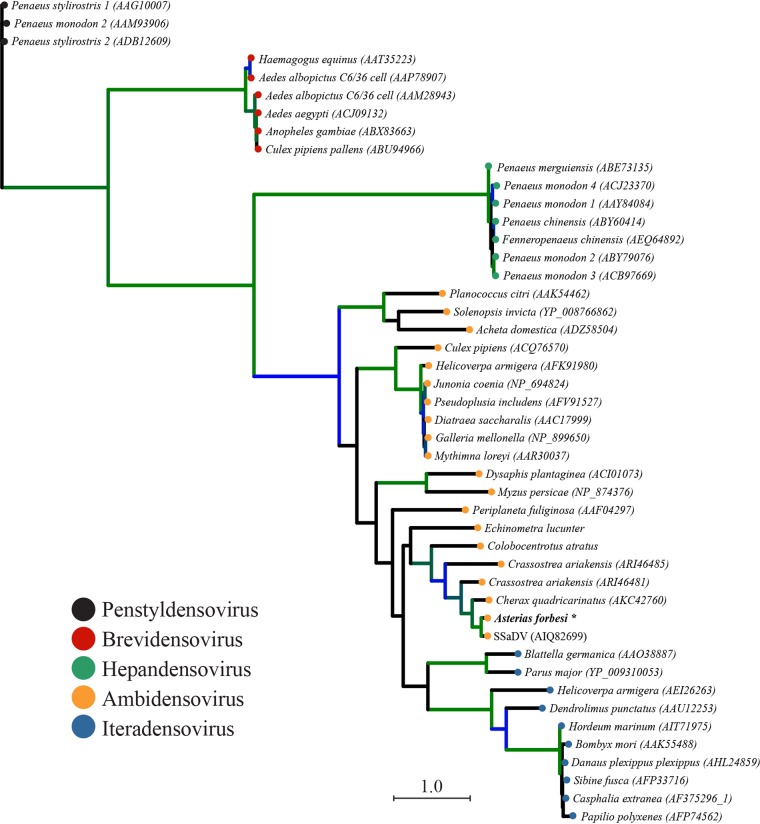
Maximum likelihood phylogeny of densoviruses (Akaike information criterion [AIC]; LG+G+I+F). The phylogenetic tree is based on an amino acid alignment performed by MUSCLE of the NS1 region spanning motif I of the RC endonuclease domain to motif C of the SF3 helicase domain (amino acid sequence length, 437.7 ± 50 [mean ± SD]). Branch supports were bootstrapped at 100 iterations and are shown as colored branches. Black branches indicate <80% support, blue branches indicate 80 to 90% support, and green branches indicate 90 to 100% support. Terminal node colors correspond to densovirus genera. Italicized names correspond to the animal genus and species from which the densovirus was isolated. AfaDV is indicated in boldface type with *.

High-throughput sequencing of metaviromes prepared from the pyloric caeca and body wall of sea star samples generated 2.42 × 10^7^ and 7.66 × 10^6^ reads/library, respectively, totaling 3.18 × 10^7^ reads. SPAdes assembly and annotation of contigs to the curated ssDNA database resulted in one contig of 6,089 nucleotides (nt) that was significantly (E value of <1 × 10^−8^) similar to SSaDV (Fig. 1). Read mapping to this contig recruited 16,737 reads that gave an average base coverage of 532, which proportionately made up 0.052% of the total reads (Fig. 1). A total of 16,732 mapped reads came from the metavirome prepared from the pyloric caeca, and 5 mapped reads came from the metavirome prepared from body wall tissue. The contig contained 4 open reading frames (ORFs) that putatively encode nonstructural proteins (NS1, NS2, and NS3) and a structural protein (VP) (Fig. 1). As a whole, the contig encodes all components of the NS and VP cassettes that are characteristic of the genus *Ambidensovirus* within the subfamily *Densovirinae*. Phylogenetic analyses (maximum likelihood) of the above-mentioned sequence indicate that this novel densovirus falls within a well-supported clade that includes other ambidensoviruses and shares a most recent common ancestor with the previously described sea star densovirus SSaDV (Fig. 2; see also Fig. S2 in the supplemental material). AfaDV and SSaDV share 77.9% pairwise nucleotide identity across their entire genomes, although the putative NS1, NS2, and NS3 genes have pairwise nucleotide identities of 88.7%, 88.1%, and 74.4%, respectively, while the putative VP genes share 77.1% pairwise nucleotide identity. According to Cotmore et al., parvoviruses of the same species encode NS1 proteins with >85% pairwise amino acid sequence identity (17). The pairwise amino acid sequence identity of NS1 between AfaDV and SSaDV is 86.5%, which makes AfaDV a new isolate of the same densovirus species. It is likely that the contig is a complete genome because of the presence of hairpins located at the ends of the inverted terminal repeats (ITRs), which are characteristic of parvoviruses (18). However, it is possible that the ITRs were not fully completed *in silico* based on the observation that ambidensovirus ITR nucleotide lengths are typically >500 nt, which raises the possibility that this genome is not complete (1). Nevertheless, the lengths of the ITRs are 271 nt, and the terminal nucleotides of the ITRs form canonical hairpin structures that are 93 nt long, which are thermodynamically favorable (Δ*G* = −52.71) (Fig. 1).

### Tissue tropism, prevalence, and biogeography.

**FIG 3:**
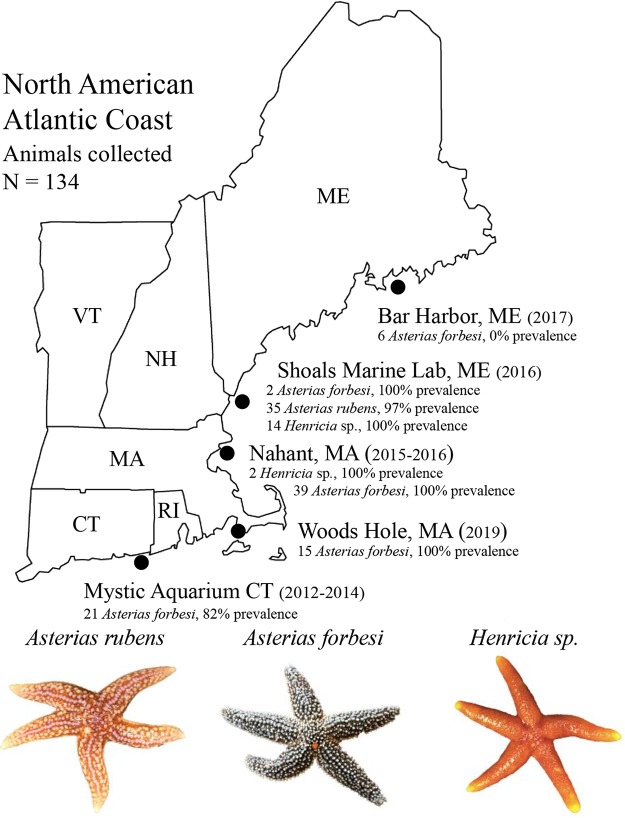
AfaDV prevalence among sea star populations along the North American Atlantic Coast (https://www.jing.fm/iclipt/u2q8u2q8i1w7t4o0/). A total of 134 animals from three species of sea stars were screened via qPCR or PCR for AfaDV. The year(s) of sampling is shown in parentheses. The prevalence for each species corresponds to the number of animals positive for AfaDV divided by the total number of animals listed next to each species.

**FIG 4:**
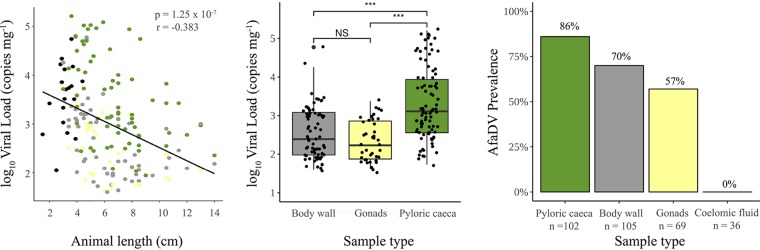
Viral load and tissue prevalence of AfaDV. (Left) Pearson’s correlation between viral load and animal length reported as total diameter. Colors correspond to sample types. Black dots represent cross-section samples. (Middle) Viral load comparison between tissue types. (Right) Prevalence of AfaDV among tissue types. ***, *P* ≤ 0.001; NS, no significance.

SSaDV was not detected in any of the pyloric caecal samples screened. AfaDV was detected from sea stars collected from 2012 to 2019 from four of the five locations (Shoals Marine Laboratory, ME; Nahant, MA; Woods Hole, MA; and the Mystic Aquarium, CT) (Fig. 3). AfaDV was not detected from 6 *Asterias forbesi* sea stars collected from Bar Harbor, ME (Fig. 3). The overlapping PCR primer set successfully amplified AfaDV sequences from the three sea star species collected in this study. To assess tissue tropism, we screened pyloric caeca, gonads, body wall, and coelomic fluid from animals via qPCR and reverse transcriptase PCR (RT-PCR). AfaDV was detected more frequently in the pyloric caeca (86%) than in the body wall (70%) and gonads (57%) and was not detected in any coelomic fluid samples (Fig. 4). AfaDV log_10_-transformed viral loads (copies mg^−1^) were significantly different among tissue types (*F*_2,197_ = 29.11; *P* = 8.38 × 10^−12^ [analysis of variance {ANOVA}]) (Fig. 4). The AfaDV log_10_-transformed viral load (copies mg^−1^) was significantly higher in the pyloric caeca (3.3 ± 0.94 [mean ± standard deviation {SD}]) than in the body wall (2.6 ± 0.66) (*P* = 5.6 × 10^−8^ [Games-Howell test]) and gonads (2.3 ± 0.52) (*P* = 5.2 × 10^−11^ [Games-Howell test]). The AfaDV load in gonads was not significantly different from that in the body wall (*P* = 0.13 [Games-Howell test]). Viral load was also significantly correlated with animal length (*P* = 1.256 × 10^−7^ [Pearson’s correlation]) and had a negative association with animal length (*r* = −0.383) (Fig. 4). Average DNA concentration differences found between tissue types did not reflect the trends found in viral loads across tissue types (Fig. S3). RT-PCR was performed on RNA extracted from pyloric caeca, gonads, and body wall samples to determine the transcription of AfaDV in these tissues. Viral transcripts were detected in all three tissue types (Fig. S4).

### Vertical transmission of AfaDV.

The pyloric caeca of 15 *Asterias forbesi* sea stars from Woods Hole, MA, were virus positive via PCR. Five out of ten oocytes isolated from females were virus positive, and 2/5 gonadal tissue samples from male sea stars were virus positive (Fig. S5).

## DISCUSSION

The discovery of SSaDV and its detection in sea stars from the Pacific and Atlantic coasts of North America suggested that SSaDV is associated with SSWS in disparate geographic regions (11). Subsequent investigations (15, 16) have supported an association between the occurrence of SSaDV and the incidence of SSWS among sea stars in the Atlantic using primers that were presumably specific for SSaDV. Previous documentation of SSaDV in Atlantic sea stars, however, may be confounded by spurious amplification and/or the presence of a genetically similar densovirus. Indeed, the nucleotide similarity of AfaDV and SSaDV suggests that primers used in other studies (11, 15, 16) may have been insufficient in distinguishing these two genotypes, which led to the conclusion that SSaDV is associated with sea stars on the Atlantic Coast. By validating the specificity of our primers, we tested the presence of both genotypes and did not find evidence for the presence of SSaDV in sea stars on the Atlantic Coast. These results suggest that SSaDV is limited to sea stars in the Pacific Northwest, which implies that any correlation with SSWS outside this region is unlikely. Although SSWS is broadly defined and is most notably observed in the Pacific Northwest, it has been observed in the South Shetland Islands (near Antarctica), in southern Australia, and in the Yellow Sea, China (19). If densoviruses are correlated with SSWS in disparate geographic regions, a unique densovirus genotype(s) may also exist in these regions. Further efforts to document the diversity and biogeography of these viruses may help to elucidate these correlations.

Currently, it is unclear what environmental or host-specific factors shape the biogeography of AfaDV and SSaDV, but the diversity and prevalence of these viruses among wild populations may be underappreciated. Similar to SSaDV, AfaDV is not associated with one species and can be found across a large geographic range (Fig. 3). To accurately document the prevalence of AfaDV, we first investigated tissue tropism, which has not been established for this virus-host system, to identify the best tissue type for viral detection (Fig. 4). Densoviruses typically have a wide tissue tropism in arthropods and can actively replicate in most tissues, although replication can be exclusively limited to certain tissues depending on the viral genotype. For example, densoviruses in the genus *Iteradensovirus* replicate exclusively and/or predominantly in midgut epithelium cells of their hosts, while Galleria mellonella and Junonia coenia ambidensoviruses replicate in almost all tissues except the midgut epithelium (1, 20). Using qPCR, PCR, and RT-PCR, we detected AfaDV in the pyloric caeca, gonads, and body wall with various degrees of prevalence, but no positive detection was found in the coelomic fluid (Fig. 4; see also Fig. S4 in the supplemental material). Comparative analysis across tissue types showed significantly higher viral loads per unit sample weight in the pyloric caeca than in other tissue types (Fig. 4). This difference was not found to be a reflection of DNA concentrations between tissue types, which suggests that the differences in viral loads across tissue types are due to a biological difference between tissue types (Fig. S3). It should be noted that our investigation of tissue tropism did not include the stomach, intestine, and radial nerves. Analysis of these tissues would be needed to establish complete tissue tropism in addition to using microscopy techniques (*in situ* hybridization or immunohistochemistry) to further validate these results. Because viral transcripts were detected in the pyloric caeca, gonads, and body wall, the differences in viral loads might result from the susceptibility of cell types, rates of cellular division among tissues, or the accessibility of the tissue to the host immune system. Given that parvoviruses replicate during the S phase of the cell cycle, the trends in viral loads across tissues likely reflect differences in cellular proliferation between these tissues (18). We hypothesize that the pyloric caecum has a larger proportion of dividing cells than other tissues, thereby explaining the observed differences in viral loads. Similarly, the correlation between viral load and animal length could be a reflection of a greater proportion of cellular division in growing individuals.

The detection of AfaDV in the same populations over a 2- to 3-year time span suggests that sea stars can maintain persistent densovirus infections. Persistent infections are common among vertebrate parvoviruses and densoviruses (21–24). Parvoviruses replicate passively and are generally more virulent in fetal and juvenile organisms, while adults can maintain persistent infections without showing any clinical signs. For example, shrimp densoviruses can cause acute infections in juveniles, but individuals that survive can carry the virus for life, transmitting it vertically and horizontally (25). Infected adults rarely show signs of disease, even in individuals with heavy infections (24). Persistent infections have been reported in other virus-host invertebrate systems. Notably, viruses that infect Apis mellifera, the European honey bee, form persistent infections that can lead to acute infections under certain conditions (26, 27). The persistence of AfaDV among host populations brings into question the capacity of sea star densoviruses to cause acute infections under particular circumstances. Such circumstances might include one or a combination of biotic and abiotic factors such as the nutritional status of the host, temperature fluctuations, microbial dysbiosis, or the presence of a vector (e.g., Varroa destructans). Considering the high prevalence of AfaDV or SSaDV among sea stars with no obvious signs of infection, the association with SSWS, if any, may be the result of a combined effect of viral infection(s) and biotic or abiotic changes.

The presence of AfaDV in DNA extracted from sea star oocytes suggests that AfaDV can infect the animal’s germ line cells (Fig. S5). Active or latent infection during embryonic development, larval growth, and metamorphosis could facilitate vertical transmission, an efficient mechanism to achieve high prevalence in adults. Further experimental and microscopic evidence will be necessary to fully establish this route of transmission. The observation of AfaDV in oocytes also suggests that these cells may be permissive. Because the whole genome of AfaDV was recovered *in silico*, a synthetic clone could be constructed and microinjected into developing embryos to test the permissiveness of these cells. Given that echinoderms are model organisms in developmental biology and infectious clones are routinely used to study densovirus biology, the tractability of this approach is promising (28–33). These experiments are currently being tested using an SSaDV clone as part of an ongoing investigation to further understand the relationship of densoviruses to sea stars.

Here, we report the discovery of a novel sea star densovirus from *Asterias forbesi*. Phylogenetic analysis demonstrates that this virus is closely related to the previously discovered sea star densovirus SSaDV. Our investigation did not find evidence for the presence of SSaDV in specimens from the North Atlantic, suggesting that SSaDV is limited to Pacific Northwest sea stars. PCR-based approaches were used to investigate the tissue tropism, prevalence among healthy sea star populations in the environment, and potential vertical transmission of AfaDV. We found AfaDV to have a broad geographic range that spreads across Connecticut to Maine and is found at a high prevalence among populations. The vertical transmission of AfaDV may explain these high prevalence rates. The results of this study further our understanding of the association between densoviruses and echinoderms beyond the context of disease. The prevalence and pervasiveness of AfaDV among wild populations suggest that these viruses might form commensal or mutualistic relationships with their hosts. The pathogenicity and interaction of AfaDV at the cellular, larval, and adult stages cannot be inferred from these data alone, but it appears that densoviruses may be common constituents of these animals’ microbiome.

## MATERIALS AND METHODS

### Viral metagenomic preparation and bioinformatic analysis.

Viral metagenomes were prepared using a protocol that was adapted and modified from existing laboratory protocols (34). The viral metagenomes used in this study were previously analyzed and reported for the presence of circular ssDNA viruses (35). Six *Asterias forbesi* sea stars that displayed signs characteristic of SSWS (arm detachment, mucoid appearance on the aboral surface, and disintegration of epidermal tissue) were collected from Canoe Beach, Nahant Bay, MA (42.420889, −70.906416) from September to October 2015 (36). Animals were flash-frozen in liquid nitrogen upon collection and then stored at −80°C until dissection. The pyloric caeca and body wall tissue from these animals were pooled separately in 0.02-μm-filtered 1× phosphate-buffered saline (PBS) and then homogenized in a bleach-cleaned NutriBullet instrument for 60 s. Tissue homogenates were pelleted by centrifugation at 3,000 × *g* for 180 s, and the supernatant was syringe filtered through Millipore Sterivex-GP 0.22-μm polyethersulfone filters into bleach-treated and autoclaved Nalgene Oak Ridge high-speed centrifugation tubes. Filtered homogenates were adjusted to a volume of 35 ml by adding 0.02-μm-filtered 1× PBS, amended with 10% (wt/vol) polyethylene glycol 8000 (PEG 8000), and precipitated for 20 h at 4°C. Insoluble material was then pelleted by centrifugation at 15,000 × *g* for 30 min. The supernatant was decanted, and pellets were resuspended in 1 ml of 0.02-μm-filtered nuclease-free H_2_O. The samples were treated with 0.2 volumes (200 μl) of CHCl_3_, inverted three times, and incubated at room temperature for 10 min. After a brief centrifugation, 800 μl of the supernatant was transferred into a 1.5-ml microcentrifuge tube. Samples were treated with 1.5 μl of Turbo DNase (2 U/μl) (Invitrogen), 1 μl of RNase One (10 U/μl) (Thermo Scientific), and 1 μl of Benzonase nuclease (≥250 U/μl) (MilliporeSigma) and incubated at 37°C for 3 h. A total of 0.2 volumes (160 μl) of 100 mM EDTA was added to the sample after incubation. Viral DNA was extracted from 500-μl subsamples using the Zymo Research viral DNA kit according to the manufacturer’s protocol and subsequently amplified isothermally at 30°C using a Genomiphi whole-genome amplification kit (GE Healthcare, Little Chalfont, UK). Samples were cleaned and concentrated using a Zymo Research DNA clean and concentrator kit, and DNA was quantified using a Quant-iT PicoGreen double-stranded DNA (dsDNA) assay kit (Invitrogen). Samples were prepared for Illumina sequencing using the Nextera XT DNA library preparation kit (Illumina, San Diego, CA, USA) prior to 2- by 250-bp paired-end Illumina MiSeq sequencing at the Cornell University Core Laboratories Center (Ithaca, NY, USA).

Libraries generated from both samples (pyloric caeca and body wall) were first interleaved into one file. Reads were then trimmed for read quality and Illumina adapters, filtered for phiX contamination, and then merged and normalized to a target depth of 100 and a minimum depth of 1 with an error correction parameter. Read quality filtering, trimming, contamination removal, merging, normalization, and read mapping were done using the BBtools suite (37). The merged and unmerged read-normalized libraries were used for *de novo* assembly using SPAdes (38). Contigs of less than 500 nt were discarded after assembly, and the remaining contigs were subjected to a tBLASTx search against a curated in-house database of ssDNA viruses (39). Contigs with significant (E value of <1 × 10^−8^) sequence similarity to SSaDV (“sea star-associated densovirus”) were isolated, and reads were mapped back to contigs with a minimum identity of 0.95 to obtain average coverage and coverage distribution across the contigs (Fig. 1). ORFs were defined in Geneious with a minimum size of 550 nt, and the hairpin structures in the inverted terminal repeats (ITRs) were determined using Mfold (40, 41).

Phylogenetic relationships among AfaDV and 45 densovirus genomes were inferred by a maximum likelihood method, implementing SMS (smart model selection) in PhyML 3.0 using a MUSCLE amino acid sequence alignment of NS1 with default parameters (42, 43). The region of NS1 used for alignment (amino acid sequence length of 437.7 ± 50 [mean ± SD]) spanned motif I of the RC endonuclease domain to motif C of the SF3 helicase domain. Branch support was determined by bootstrapping at 100 iterations. The phylogenetic tree was visualized and edited using iTOL (44).

### Specimen collection, nucleic acid extraction, and cDNA synthesis.

Three species of sea stars, *Asterias forbesi*, Asterias rubens, and *Henricia* sp., were collected from five locations along the Atlantic Coast of the United States from 2012 to 2019 (see Table S1 in the supplemental material) (36). Eighty-three *Asterias forbesi*, 16 *Henricia* sp., and 35 *Asterias rubens* sea stars were collected, totaling 134 sea stars. Prior to vivisection or dissection, animal length was measured by total diameter (i.e., ray to ray). Animals were either vivisected immediately after collection, flash-frozen in liquid nitrogen upon collection, and then stored at −80°C until dissection or cryopreserved and stored at −20°C until dissection. Coelomic fluid was extracted from only vivisected animals using a 25-gauge 1.5-in. (0.5-mm by 25-mm) needle attached to a 3-ml syringe inserted through the body wall into the coelomic cavity. Gonads, body wall, and pyloric caeca were collected from all vivisected animals, but not every tissue type was collected from animals that were dissected (Table S1). In total, 368 samples were collected. DNA was extracted from tissues (14 to 200 mg [wet weight]) and coelomic fluid samples (140 μl to 1,000 μl) using Zymo Research tissue and insect DNA kits or a Zymo Research quick DNA miniprep plus kit according to the manufacturer’s protocol (Table S1). All DNA samples used for qPCR were extracted with the Zymo Research tissue and insect DNA kit. Coelomocytes in the coelomic fluid were pelleted by centrifugation at 10,000 × *g* for 5 min and then resuspended in 200 μl of 0.02-μm-filtered nuclease-free H_2_O prior to DNA extraction. DNA was quantified using the Quant-iT PicoGreen dsDNA assay kit (Invitrogen). RNA was extracted from pyloric caeca, gonads, and body wall samples from *Asterias forbesi* collected from Nahant, MA, using the Zymo Research tissue and insect RNA microprep kit. An in-column DNase I digestion step was performed according to the manufacturer’s protocol. The Maxima first-strand cDNA synthesis kit (Thermo Scientific) was used for cDNA synthesis following RNA extraction. Four microliters of eluted RNA from each sample was stored at −80°C and was used as a no-reverse transcriptase (RT) control for RT-PCR analysis.

### Genome verification.

AfaDV was amplified by PCR with overlapping primers to produce 16 amplicons. Amplicons generated from PCR were gel visualized with ethidium bromide, and the remaining PCR product was cleaned and concentrated using a Zymo Research DNA clean and concentrator kit and submitted for Sanger sequencing at the Cornell University Core Laboratories Center (Ithaca, NY, USA). The resulting amplicons were assembled to form a contig spanning nucleotide positions 214 to 5860 (92.7% of the total genome length). The assembled contig was identical to the contig generated from the *de novo* assembly. This process was repeated on tissue samples from the three collected sea star species to verify the viral genome sequences among each host species. Primers were designed using Primer3 (45). Reaction conditions and primer sequences can be found in Table 1.

**TABLE 1:** Primer/probe sequences, thermocycler parameters, and purpose for each PCR assay

Purpose and oligonucleotide, probe, or primer	Sequence^*a*^	Thermocycling parameters
AfaDV quantification (qPCR)		
Standard	TTCGTAATAGCACCTTCGTCACCAGCTAAATATAGATTTTCTCCAACTCTGATGAAACCAGCACGTCCACCAGACTGATGAATGCGCAGTAATTGTCTA	1 cycle at 50°C for 2 min, followed by 95°C for 2 min and 50 cycles of 95°C for 15 s and 58°C for 1 min (SsoAdvanced universal probes supermix used)
Internal probe	FAM-TGATGAAACCAGCACGTCCACCAGA-TAMRA	
L-primer position 5664	CGTAATAGCACCTTCGTCACC	
R-primer position 5738	GACAATTACTGCGCATTCATCA	
AfaDV genome verification (PCR)		
L-primer 1 position 243	TTTGAGGTCATATGGGCGGA	1 cycle at 94°C for 2 min; 30 cycles of 45 s at 94°C, 30 s at 56°C, and 45 s at 72°C; and a final extension step for 2 min at 72°C (*Taq* DNA polymerase used)
R-primer 1 position 833	CTTGTCACAACTCCTTTTCGC	
L-primer 2 position 554	ACGCCACTCAGTATGCAGTA	
R-primer 2 position 1061	TCCCAAGCTTTGCCAGAGTA	
L-primer 3 position 831	TGCGAAAAGGAGTTGTGACA	
R-primer 3 position 1361	TGCAAACGCTATCTTCTTCTCC	
L-primer 4 position 1266	TGCCGGATCTGACCATTGAT	
R-primer 4 position 1702	TTCTCGACATACCTGGAGCA	
L-primer 5 Position 1520	AAGCAGCAAAGACATGGAGC	
R-primer 5 position 2075	GATCCGGTTCGTCATCATCG	
L-primer 6 position 1979	GGAGAGCGGACTTGATGGAT	
R-primer 6 position 2564	AGAAATTCTTACCCGCTGAAGG	
L-primer 7 position 2378	GTGCAGGGTACGGTAATTTTG	
R-primer 7 position 2919	ACAGCAAGCGGATTAGGTTTC	
L-primer 8 position 2655	CCATTTCAAGACGCTGAGGG	
R-primer 8 position 3144	AATGTTGCTCCACCAGTTGC	
L-primer 9 position 3066	CTTGGGCGAGTCATACGAGA	
R-primer 9 position 3599	AGTCTGTTGGAAACGCTCAG	
L-primer 10 position 3440	AGCAGAGTCACCACGAACAT	
R-primer 10 position 3895	CGGTACTGATCAATCTTCTGCT	
L-primer 11 position 3707	TGATCCCAAGTAGTATCGTTCG	
R-primer 11 position 3999	ATGAGAGGAGGAGTCGATAGG	
L-primer 12 position 3895	AGCAGAAGATTGATCAGTACCG	
R-primer 12 position 4397	ATTCGCAAAGTGATGGAGGC	
L-primer 13 position 4215	TGGGATTTTAGCGAGAGGAGT	
R-primer 13 position 4770	AGATCACGTCCTAGTAGTGCT	
L-primer 14 position 4582	CACCTTCAGCTTGGCGTATA	
R-primer 14 position 5031	TCTTCCTCAGGTATGTCGCA	
L-primer 15 position 4914	TGTTGGCCCTTTTGAGTAGG	
R-primer 15 position 5461	TGTTGCTGCTGGTACTTCTG	
L-primer 16 position 5257	TCGTCATCAACATCAACAGGC	
R-primer 16 position 5866	TTTGAGGTCATATGGGCGGA	
AfaDV oocyte and pyloric caecum detection, VP mRNA (PCR/RT-PCR)		
L-primer 9 position 3066	CTTGGGCGAGTCATACGAGA	1 cycle at 98°C for 30 s; 35 cycles of 10 s at 98°C, 20 s at 66°C, and 20 s at 72°C; and a final extension step for 2 min at 72°C (Q5 polymerase used)
R-primer 9 position 3599	AGTCTGTTGGAAACGCTCAG	
AfaDV VP cloning (PCR)		
L-primer position 252 (restriction enzyme EcoRI)	CGCgaattcATAGAAAAGGCTGTG	1 cycle at 98°C for 30 s; 30 cycles of 10 s at 98°C, 30 s at 66°C, and 1 min 30 s at 72°C; and a final extension step for 2 min at 72°C (Q5 polymerase used)
R-primer position 3187 (restriction enzyme HindIII)	CGCaagcttCCTAATCCGCT	
SSaDV VP cloning (PCR)		
L-primer position 2857 (restriction enzyme HindIII)	GGGGaagcttAGAAACCTAATCC	1 cycle at 98°C for 30 s; 30 cycles of 10 s at 98°C, 30 s at 67°C, and 1 min 30 s at 72°C; and a final extension step for 2 min at 72°C (Q5 polymerase used)
R-primer position 5731 (restriction enzyme EcoRI)	CTGAgaattcCATTATGTCGGGTG	
AfaDV NS1, NS2, and NS3 cloning (PCR)		
L-primer 1 position 243	TTTGAGGTCATATGGGCGGA	1 cycle at 98°C for 30 s; 30 cycles of 10 s at 98°C, 30 s at 67°C, and 1 min 30 s at 72°C; and a final extension step for 2 min at 72°C (Q5 polymerase used)
R-primer 8 position 3144	AATGTTGCTCCACCAGTTGC	
SSaDV (PCR)		
SSaDV_NS3_1_F	CAATACGCCGATTAGCTTACAG	1 cycle at 98°C for 30 s; 35 cycles of 10 s at 98°C, 30 s at 64°C, and 40 s at 72°C; and a final extension step for 2 min at 72°C (Q5 polymerase used)
SSaDV_NS2_1_R_2	TCCTCGCTCACTACTAATGTTG	

a Lowercase type in sequences indicates the restriction enzyme. FAM, 6-carboxyfluorescein; TAMRA, 6-carboxytetramethylrhodamine.

### Cloning.

Putative VP and NS genes of AfaDV and SSaDV were inserted into the pGEM-T Easy vector and used to transform NEB 5-alpha competent cells. Primers, PCR conditions, and plasmid constructs can be found in Table 1 and Fig. S1. PCR amplicons were gel purified, poly(A) tailed, and ligated to pGEM-T Easy vectors. The pUC19-SSaDV construct was synthesized by GenScript. Plasmid constructs were verified by Sanger sequencing.

### AfaDV prevalence and viral load.

Viral prevalence and viral load were determined by quantitative PCR (qPCR) using TaqMan chemistry. The qPCR primers and probe were designed using Primer3 targeting the VP region spanning positions 5634 to 5728 (45). Reaction conditions and primer/probe/oligonucleotide standard sequences can be found in Table 1. All qPCRs, including no-template controls, were performed in duplicate on a StepOnePlus real-time PCR system (Applied Biosystems). A synthetic oligonucleotide sequence spanning the qPCR primer/probe region was used to generate the standard curve using duplicate 8-fold serial dilutions, which were included in all qPCR runs. Twenty-five-microliter reaction mixture volumes contained 0.02 μl (200 pmol) of each primer and probe, 12.5 μl of 2× SsoAdvanced universal probe supermix (Bio-Rad), 10.44 μl of nuclease-free H_2_O, and 2 μl of the template. Viral quantity was calculated using StepOnePlus software (version 2.3; Applied Biosystems) by averaging the cycle threshold (*C_T_*) values between duplicates and interpolating values against the standard curve. Viral quantities were adjusted by extraction volume and standardized by sample weight. Samples were considered positive when both technical replicates were positive and the *C_T_* standard deviation was <1.0. The lower limit of detection used in this study was 30 copies per reaction (average *C_T_* value of 34) or 40 copies·mg^−1^ tissue. Viral prevalence was defined as the total number of positive samples for a sample type, and viral load was defined as the mean copy number from technical replicates in a positive sample.

### Oocyte collection, DNA extraction, PCR, and SSaDV survey.

Fifteen *Asterias forbesi* sea stars were collected from Woods Hole, MA, from June to July 2019. Gonadal tissue and pyloric caeca were taken through a small incision in the arm and manually extracted with forceps (Table S1). Isolation of oocytes was performed according to methods described previously by Wessel et al. (28). Oocytes were isolated by mincing gonadal tissue in 0.2-μm filtered seawater, which was then poured through cheesecloth, pelleted by centrifugation, decanted, and pipetted for DNA extraction. DNA extractions were performed using a Zymo Research quick DNA miniprep plus kit. PCRs included a kit negative control (i.e., extraction blank) and a PCR reagent negative control to account for false-positive results. PCR cycle conditions can be found in Table 1.

Thirty pyloric caecal samples were screened for SSaDV to determine the presence of SSaDV among North Atlantic sea stars. Ten pyloric caecal samples from Woods Hole, Shoals Marine Lab, and Nahant were chosen. The specificity of primers was validated (i.e., no cross amplification observed) by screening against the appropriate plasmid constructs prior to screening DNA extracts (Fig. S1). PCR cycle conditions can be found in Table 1.

### Data availability.

The AfaDV genome sequence has been deposited in GenBank under accession number MN190158. Metagenomic libraries have been deposited in GenBank under BioProject accession number PRJNA555067. Data from all statistical and bioinformatics analyses can be found at https://github.com/ewj34/AfDV-Viral-Metagenome.

## Supplementary Material

Supplemental file 1

Supplemental file 2
