# Supplementary material for: A Highly Prevalent and Pervasive Densovirus Discovered among Sea Stars from the North American Atlantic Coast
Source: Appl Environ Microbiol. 2020 Mar 2;86(6):e02723-19. doi: 10.1128/AEM.02723-19 (PMC7054102; doi:10.1128/AEM.02723-19)

Supplemental Figure 1 – Plasmid constructs created for primer specificity analysis. A) Densovirus genome architecture. ORFs colored and labeled by putative function. Red represents structural proteins (VP) and blue represents non-structural proteins (NS) B) Plasmid constructs created using pGEM-t-Easy.

A)

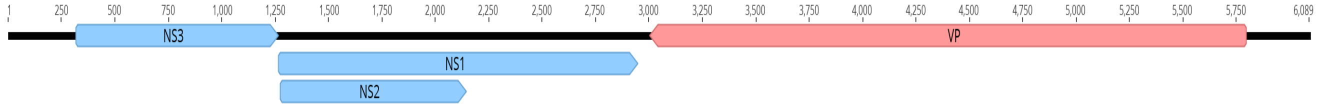

B)

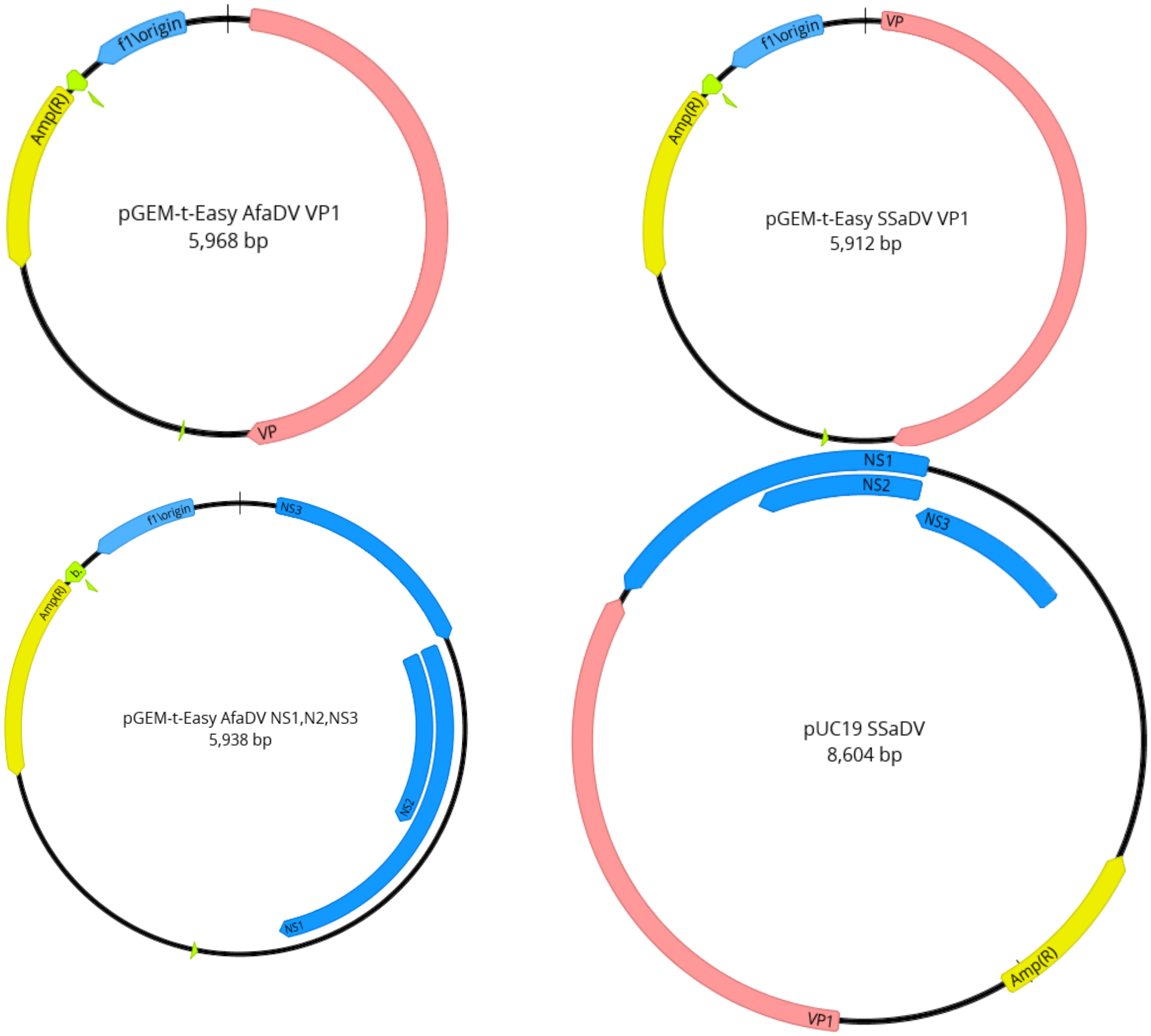

Supplemental Figure 2: Maximum likelihood phylogeny of densoviruses (AIC; LG +G+I+F). Cladogram is based on an amino acid alignment performed by MUSCLE of the NS1 region spanning Motif I of the RC endonuclease domain to Motif C of the SF3 helicase domain (amino acid sequence length 437.7 ± 50; mean ± SD). Branch support bootstrapped at 100 iterations and terminal node colors correspond to densovirus genus. Italicized names correspond to animal genus and species for which the densovirus was isolated. AfaDV denoted in bold with *.

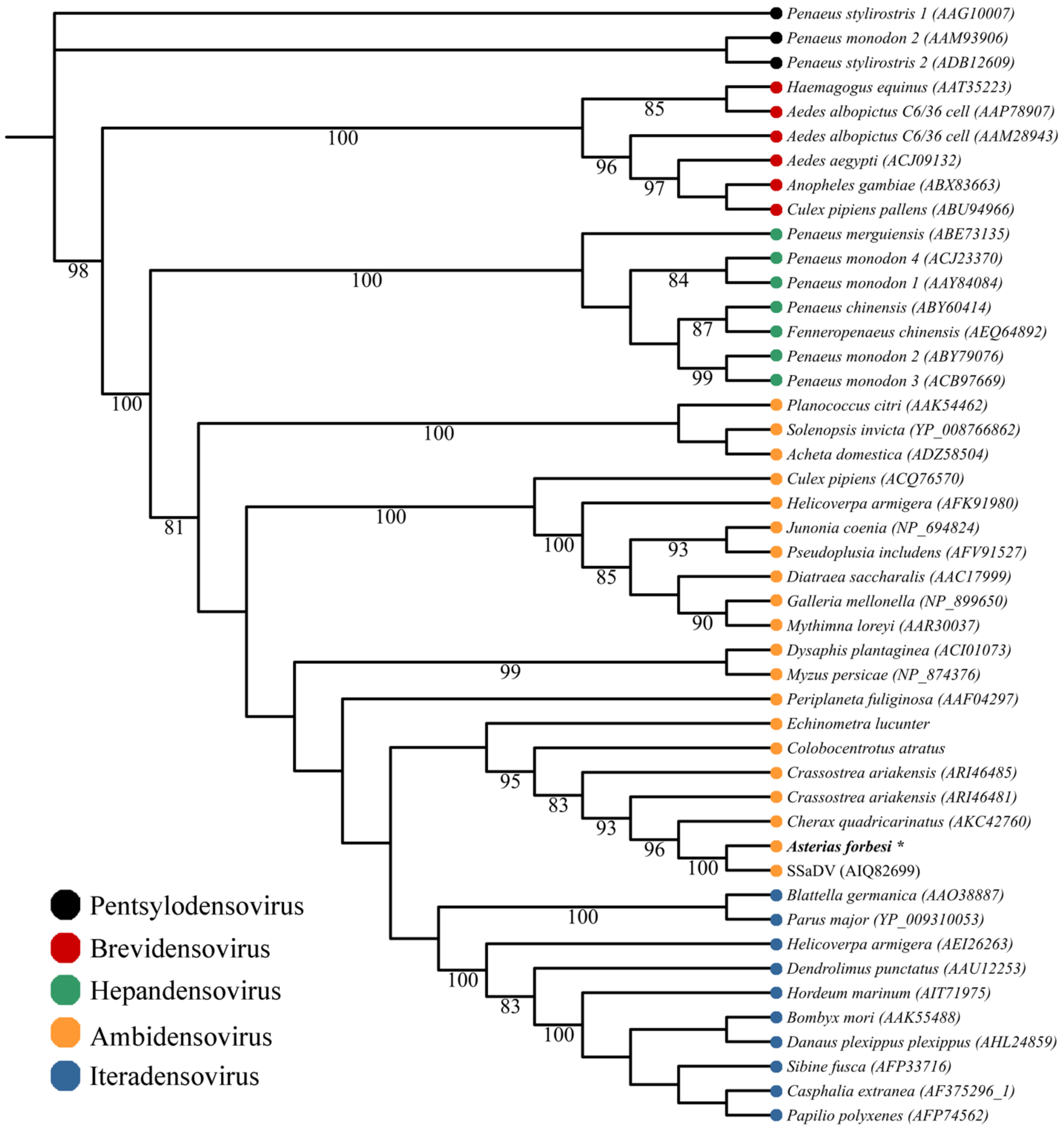

Supplemental Figure 3: Mean DNA concentration across samples types. Error bars represent two standard errors from the mean.

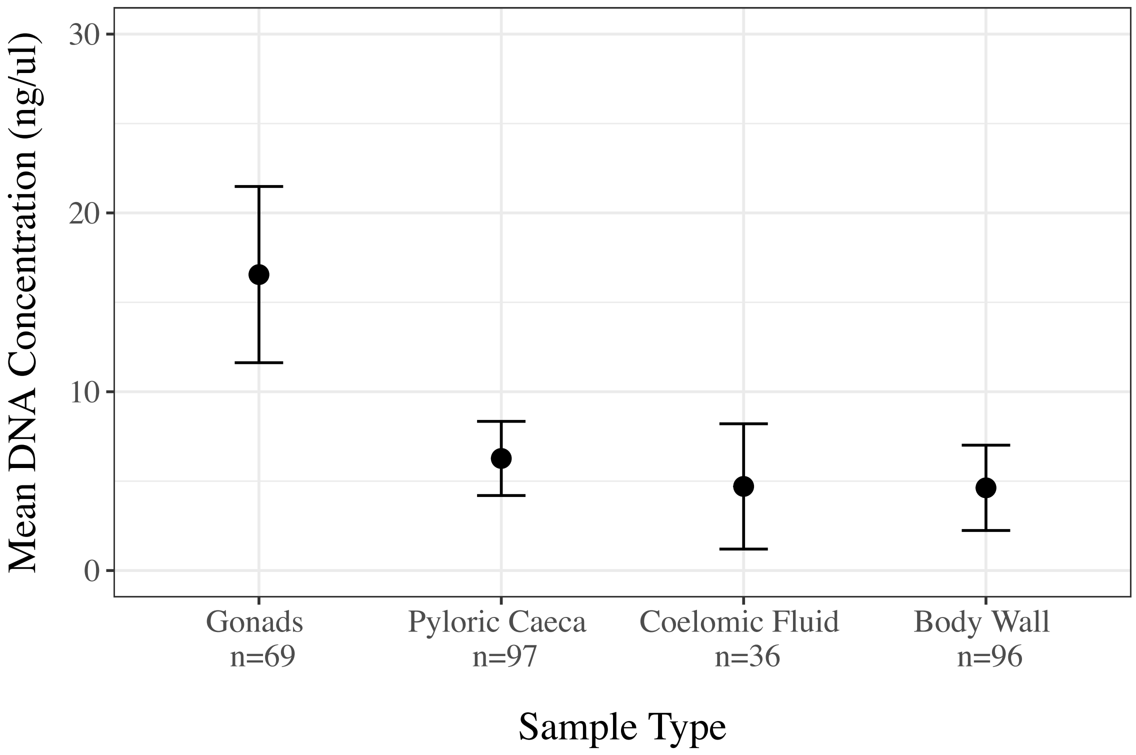

Supplemental Figure 4: RT-PCR detection of AfaDV from RNA extracted from pyloric caeca, body wall, and gonads from *Asterias forbesi.* A = cDNA template. B = RNA with no-reverse transcription control template. (-) = kit extraction blank. (+) = PCR positive control using cloned AfaDV VP plasmid.

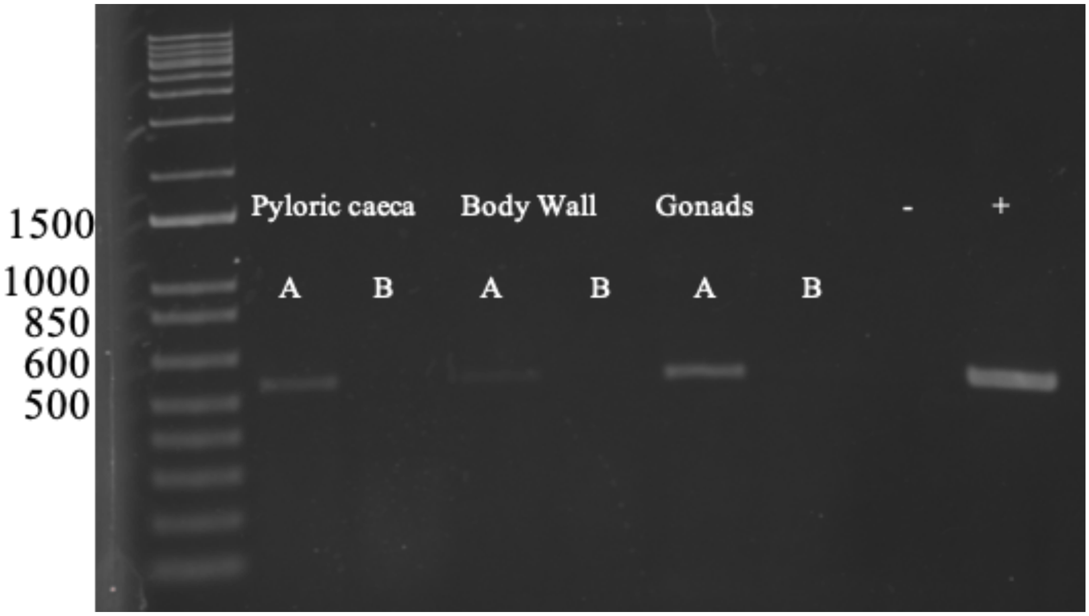

Supplemental Figure 5: PCR detection of AfaDV from DNA extracted from pyloric caeca and oocytes collected from 10 female *Asterias forbesi* from Woods Hole, Massachusetts. B = kit extraction blank for non-template control PCR.

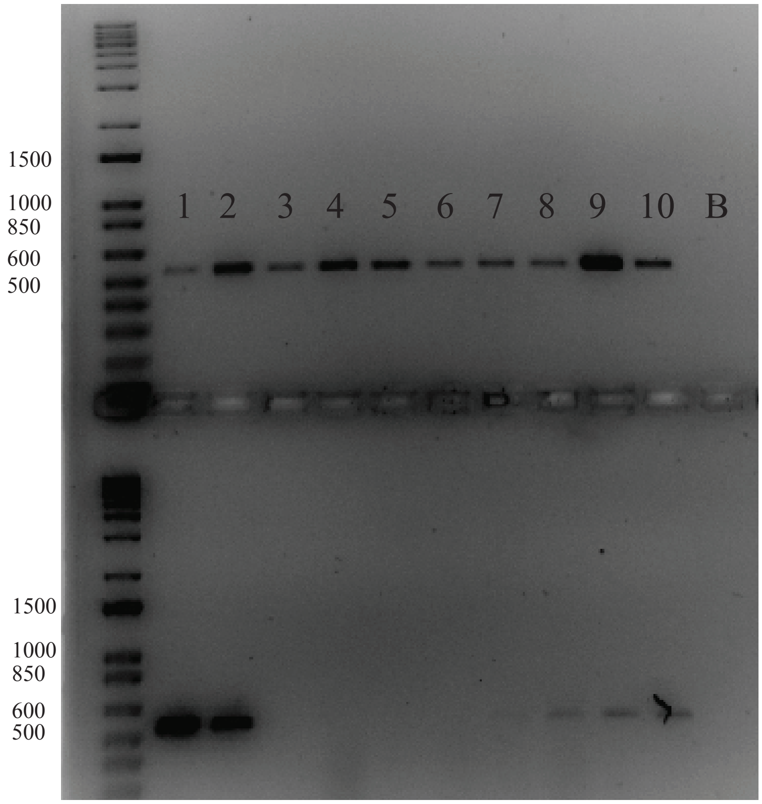

Supplement: Supplemental file 1 [file AEM.02723-19-s0001.pdf]
